# The Early Activation of Toll-Like Receptor (TLR)-3 Initiates Kidney Injury after Ischemia and Reperfusion

**DOI:** 10.1371/journal.pone.0094366

**Published:** 2014-04-15

**Authors:** Patrick Paulus, Katrin Rupprecht, Patrick Baer, Nicholas Obermüller, Daniela Penzkofer, Christin Reissig, Bertram Scheller, Johannes Holfeld, Kai Zacharowski, Stefanie Dimmeler, Joelle Schlammes, Anja Urbschat

**Affiliations:** 1 Department of Anesthesiology, Intensive Care Medicine and Pain Therapy, Goethe-University Hospital Frankfurt, Frankfurt am Main, Germany; 2 Department of Internal Medicine III, Division of Nephrology, Goethe-University Hospital, Frankfurt am Main, Germany; 3 Institute of Cardiovascular Regeneration, Goethe-University, Frankfurt am Main, Germany; 4 Department of Cardiac Surgery, Innsbruck Medical University, Innsbruck, Austria; 5 Faculty of Medicine, Philipps-University Marburg, Marburg, Germany; National Cancer Institute, United States of America

## Abstract

Acute kidney injury (AKI) is one of the most important complications in hospitalized patients and its pathomechanisms are not completely elucidated. We hypothesize that signaling via toll-like receptor (TLR)-3, a receptor that is activated upon binding of double-stranded nucleotides, might play a crucial role in the pathogenesis of AKI following ischemia and reperfusion (IR). Male adult C57Bl6 wild-type (wt) mice and TLR-3 knock-out (-/-) mice were subjected to 30 minutes bilateral selective clamping of the renal artery followed by reperfusion for 30 min 2.5h and 23.5 hours or subjected to sham procedures. TLR-3 down-stream signaling was activated already within 3 h of ischemia and reperfusion in post-ischemic kidneys of wt mice lead to impaired blood perfusion followed by a strong pro-inflammatory response with significant neutrophil invasion. In contrast, this effect was absent in TLR-3-/- mice. Moreover, the quick TLR-3 activation resulted in kidney damage that was histomorphologically associated with significantly increased apoptosis and necrosis rates in renal tubules of wt mice. This finding was confirmed by increased kidney injury marker NGAL in wt mice and a better preserved renal perfusion after IR in TLR-3-/- mice than wt mice. Overall, the absence of TLR-3 is associated with lower cumulative kidney damage and maintained renal blood perfusion within the first 24 hours of reperfusion. Thus, we conclude that TLR-3 seems to participate in the pathogenesis of early acute kidney injury.

## Introduction

Ischemia reperfusion (IR) injury is one of the leading causes for the clinical manifestation of acute kidney injury (AKI) and is still associated with a high mortality in the critically ill patient [1], [2]_ENREF_1. It is mainly caused by complex inflammatory processes that affect the vascular as well as the tubular system in the kidney [3]. In clinical every day-life, severe sepsis episodes and ischemic conditions following cardiac or renal surgery as well as kidney transplantation are considered as the main reasons for renal ischemia [4], [5], [6], [7].

Histomorphologically, patients with severe ischemic insults impacting their kidneys show proximal tubular necrosis. This is the result of an acute tubulo-interstitial process that is caused and amplified by cellular inflammation mainly caused by neutrophils and edema [3], [8], [9]. The mechanisms that induce the inflammatory response in the kidney and tubular cells may share a common pathway: pro-inflammatory response upon toll-like receptor (TLR) activation [9]. TLRs are key molecules of the innate immune system that mediate their signal transduction through the activation of transcription factors that regulate the expression of pro-inflammatory cytokines and chemokines [10], [11], [12]. Hence, the theory that cells subjected to hypoxia release potential TLR ligands is not new [11]. Many studies have shown the impact of TLR-2 and -4 in kidney ischemia and reperfusion [13], [14], [15]. However TLR-2/-4 seem to be activated much later than we observed for TLR-3, which is regulated within minutes following IR. So far, the role of nucleic acid binding toll-like receptors like i.e. TLR-3 has not yet been investigated in AKI. TLR-3 is a receptor that has initially been described to induce antiviral responses by recognition of viral components such as single or double-stranded RNA (ssRNA/dsRNA). Its activation results in the production of type I interferons (IFNs), and enhancement of apoptosis [11], [16]. Recently it has been shown that TLR-3 is expressed on mouse and human kidney tubular epithelial cells [17], [18]. These cells appear to express TLR-3 constitutively and upregulate its expression upon challenge with its ligands [19]. Thus it seems plausible that this receptor might play an important role in this organ in health and disease [20]. Moreover, highly active cells like epithelial, hepatic or renal cells are subjected to a high transcriptional turnover rate and thus contain high levels of cytosolic RNA.

Thus, these facts prompted us to investigate the role of TLR-3 in renal IR injury using a model of bilateral kidney IR in the mouse. In this setting, we evaluated if, or to what extent TLR-3 signaling is participating in renal ischemia and reperfusion stress.

## Materials and Methods

### Animals

Male adult C57/BL6 (Janvier, France) and TLR-3 knockout (TLR-3-/-, C57/BL6 background) mice (25–30 g) were kept in approved plastic cages, had free access to water and food and were housed in rooms equipped with a 12 h light cycle. All animal procedures were approved by the Animal Care and Use Committee of the state of Hessen, file number: V54-19c20/15-F35/04. Surgical procedures and animal care were performed in accordance with the „Guide for the care and use of laboratory animals“ (NIH, volume 25, no. 28, revised 1996), EU Directive 86/609 and German Animals Protection Act.

### Induction of bilateral kidney ischemia and reperfusion

C57/BL6 respective TLR-3 knockout mice were randomly assigned to 4 groups (each n = 6): sham-treated, 1 h (designated as: wt 1 h resp. TLR-3-/- 1 h), 3 h (wt 3 h resp. TLR-3-/- 3 h) and 24 h (wt 24 h resp. TLR-3-/- 24 h) of reperfusion. Mice were anaesthetized with an intraperitoneal injection of ketamine (100 mg/kg body weight) and xylazine (5 mg/kg body weight). During anesthesia (for surgery and for sonography), the constant core temperature of 37°C was controlled using a rectal probe and body temperature was stabilized by using a heating pad. After bilateral dorsal flank incision the kidney vessels were prepared. Renal ischemia was induced by selectively clamping of the renal arteries for 30 minutes followed by 0.5, 2.5 or 23.5 hours of reperfusion. Selective arterial clamping was performed under microscopic control using non-traumatic microvascular clamps with a jaw pressure of 85 g (Micro-Serrefine, Fine surgical instruments, Germany). To prevent arterio-venous clamping, the correct placement of the clamps was controlled microscopically. Correctly clamped kidneys turned pale, whereas arterio-venously clamped kidneys had a hemorrhagic, deep purple aspect and had to be excluded from the experiment. At the end of the ischemia-time, clamps were released and the recovery of renal blood flow was visually monitored. Subsequently, incisions were closed in layers and mice were allowed to recover in a heated surrounding. Animals were sacrificed after 1, 3 or 24 hours of completed ischemia-reperfusion according to the protocol [21].

### Color duplex sonography and renal blood flow measurements

To track renal perfusion changes during IR we measured the perioperative resistive indexes (RI) as surrogates for kidney perfusion using color duplex sonography performed with a Vevo 2100 imaging system (VisualSonics Inc., Toronto, Canada). For color duplex measurements, anaesthetized animals were first analyzed prior to the surgical intervention and then after the corresponding time-points. Each animal was controlled for heart rate using an ECG and for spontaneous breathing using a breathing detector attached to the Vevo system. Animals' body temperature was controlled as described earlier. Each study consisted in recording 10 s video sequences. Blood flow profiles were recorded from the peripheral arcuate arteries using PW-mode and synchronized to the electrocardiogram-signature. End-systolic and end-diastolic renal blood velocities were determined using the VisualSonics software. For evaluation we calculated resistive index from at least 15 flow curves per file. Resistive index was then calculated using the formula: (VO_syst._-VO_diast._)/VO_syst._ ×100%. In preoperative animals the 25% percentile of the resistive index values was 71.47% indicating the lower normal value and the 75% percentile of the resistive index values was 79.48%, indicating the upper normal value for mouse resistive index variability [21].

### Measurement of serum creatinine

Colorimetric evaluation of serum creatinine (sCrea) was performed using the alkaline picrate method (Jaffé's Method), (LT-SYS CREATININ Jaffé, kinetic LT-CR 0121, Eberhard Lehmann GmbH, Germany). Absorbance was measured at wavelength 492 nm. Values are expressed in mg/dl. Readout was performed using a spectrophotometer (Jasco V-530 UV/VIS Spectrophotometer, Germany).

### ELISA and protein expression arrays

ELISA on mouse renin and mouse aldosterone (Thermo Fisher, Germany) were performed according to the manufacturer's protocol. Detection was performed using a microplate reader (Bio-Tek Instruments, Germany). Protein arrays (R&D Systems, UK) were used to detect protein expression patterns between ko and wt mice. Arrays were performed according to the manufacturer's protocol and fluorescence detection was performed using a Kodak Imager (Carestream, Germany).

### Histopathologic examination

Paraformaldehyde-fixed (4%) and paraffin-embedded kidneys were sectioned at 5 µm thickness. Periodic acid Schiff (PAS) staining was performed according to standard protocols. Briefly, tissue slides were oxidized in 0.5% periodic acid solution for 5 minutes followed by incubation with Schiff reagent for 15 minutes until sections become light pink. After washing the slides in tap water for 5 minutes, the color turns into dark pink. Finally samples are counterstained with Mayer's hematoxylin. Histological examinations were conducted in a blinded fashion; for each slide, 3 fields at 400-times magnification were photographed from the outer stripe of outer medulla. For histomorphological examination images were taken with the Leica DM5000B microscope and analyzed using an automatized Matlab script (The Mathworks, USA) programmed by the authors. The routine determines the color-values of pixels in the red-green-blue space (RGB), thus measuring the relative area occupied by blue (cores) staining per image. Quantification was done by setting blue colored pixels in proportion to the total number of colored pixels [21], [22], [23].

### Immunofluorescence and TUNEL Assay

TUNEL-staining was performed according to the manufacturer's protocol and quantification was done with the Matlab routine as described above. For specific neutrophil (rat anti-mouse Ly-6B.2, Serotec, Germany), TLR-3 (rabbit anti-mouse TLR-3, Sigma-Aldrich, Germany) and CD13 (rat anti-mouse, AbD Serotec, Germany) staining, slides were incubated with the primary antibody overnight at 4°C. For signal detection, secondary antibodies (donkey anti-rat IgG Alexa 488, Jackson Immuno Research, USA and donkey anti-rabbit Fab'2 PE, Fitzgerald, USA) were incubated for 1 h at room temperature. Nuclear staining was performed using DAPI staining according to the manufacturer's protocol. The slides were mounted in aqueous medium and analysis of the slides was performed in a blinded fashion. For every micrograph 10 randomly chosen fields at 400-times magnification were photographed from the outer stripe of outer medulla. Neutrophils were quantified by counting the number of neutrophils per field in a blinded fashion.

### Real-time RT-PCR

Total RNA was isolated from homogenized kidney samples and cDNA was synthesized as described elsewhere. Gene expression profiles of mouse specific genes were assessed by quantitative real-time polymerase chain reaction using a StepOne Plus real-time PCR device (Applied Biosystems, USA) and procedures were performed as described in detail elsewhere [21], [22], [23], [24]. Primer sequences are listed in *table 1*.

**Table 1:** Primer Sequences.

Gene	Accession N°	forward (5′ – 3′)	reverse (5′ – 3′)
**mu 18s**	NM_011296.2	GTAACCCGTTGAACCCCATT	CCATCCAATCGGTAGTAGCG
**mu ICAM-1**	NM_010493.2	ACTGTGGCACCGTGCAGTCG	GCTCCGTGGTCCCCTCTGCT
**mu IFNβ1**	NM_010510	TCGGACCACCATCCAGGCGT	ACCTCACCTACAGGGCGGACT
**Mu CAIX**	NM_139305.2	GGTGCACCTCAGTACTGCTT	GCAGGGAAGGAAGCCTCAAT
**mu CXCR-4**	NM_009911.3	ACGGACAAGTACCGGCTGCAC	GGCCTCTGACTGTTGGTGGCG
**mu KDR**	NM_010612.2	CAGACTGTGTCCCGCAGCCG	GCGACAGCTAGCAGCGCCTT
**mu NGAL**	NM_008491.1	CCAGGGCTGGCCAGTTCACTC	TGGGTCTCTGCGCATCCCAGT
**mu P-Selectin**	NM_011347.2	ACGGTACCATGTCCCCAAGCT	CCAGCGCTCGTGGAATCTCTC
**mu Tie-2**	NM_013690.2	CATGCGAGCGGGAAGTCGCA	ATGGGCTCATGGGGGTGCCA
**mu TLR-3**	NM_126166.4	TGCTCAGGAGGGTGGCCCTT	CACCGGGGTTTGCGCGTTTC
**mu VE-Cadherin**	X83930.2	CGCGGTGGCTCCACTAAGCC	CTGCGATGGACTCTGCGCCC

### Protein Isolation and Western Blot Analysis

Proteins from frozen kidneys were isolated as described in detail elsewhere [22]. SDS-gels (10% and 7.5%) were loaded with 50 µg protein. Proteins were immunodetected on Hybond C supermembrane (Amersham Pharmacia Biotech, UK) with Spectra brood range marker (Fermentas, Germany) as a standard. The blots were probed with antibodies as follows: rabbit anti-mouse IRF3 (Thermo Fisher Scientific, Germany), rabbit anti-mouse TRIF (Thermo Fisher Scientific, Germany) and rabbit anti-mouse Caspase 3 (New England Biolabs GmbH, Germany). Detection was then performed by incubating the membranes with horseradish peroxidase–conjugated secondary antibodies (Amersham Pharmacia Biotech, UK). Proteins were immunodetected by chemiluminescence (Supersignal-West Femto, Pierce, USA). Digitalization and evaluation of the blots were performed with a Kodak Imager (Carestream, Germany).

### Statistical analysis

Statistical analysis was performed with GraphPad Prism 5.02 software (GraphPad Software, Inc., USA). We used a frequency distribution test, indicating the 25^th^ and 75^th^ percentiles as lower and upper limits of the normal values' distribution to determine the range of normal resistive index values. Results are expressed as means ± standard error of the mean (SEM). Statistical significance was calculated using one-way ANOVA followed by Bonferroni's multiple comparison test. Statistical significance was set to p<0.05.

## Results

### Ischemia and Reperfusion activates TLR-3 pathway in kidneys of wt but not TLR-3 -/- mice

**Figure 1:**
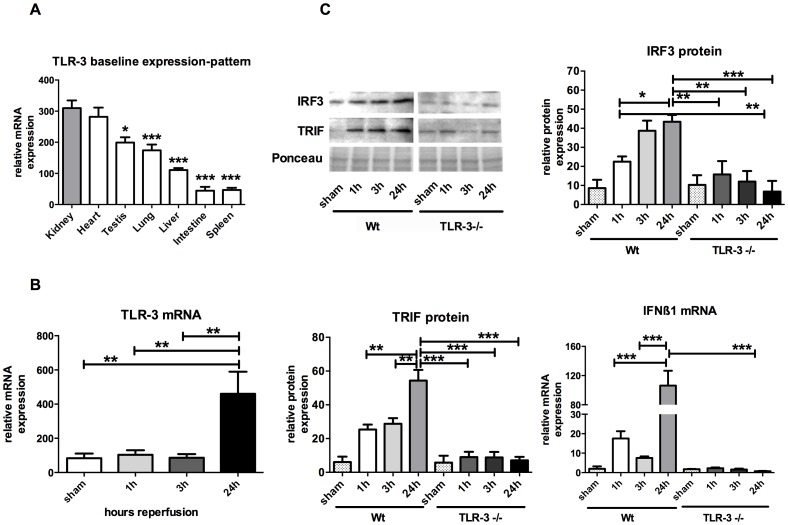
TLR-3 is regulated in vivo. TLR-3 mRNA organ expressions (A). Activation of TLR-3 during ischemia and reperfusion (B) Western Blot and RT-PCR analyses of the TLR-3 signaling pathway: IRF3, TRIF and IFN-β1 (C). (*, P<0.05; **, P<0.01; ***, P<0.001).

Among the examined organs (testis, lung liver, intestine and spleen), the basal expression level of TLR-3 was highest in the kidney (Figure 1A). In PCR analyses wild-type kidneys subjected to IR, we found that TLR-3 mRNA was significantly upregulated after 24 hours of reperfusion vs. sham treatment in wt animals (460.4±129.5 vs. 83.84±27.22 relative mRNA expression; P<0.01). These results indicate an involvement of TLR-3 signaling during IR in vivo as its respective gene expression turnover is increased. TLR-3-/- mice clearly showed a lack of TLR-3 expression (Figure 1B).

The TLR-3 pathway member interferon regulatory factor (IRF)-3 protein expression was significantly upregulated in wt mice over time (22.49±2.75 (1 h) vs. 43.45±3.56 (24 h) relative protein expression, P<0.05) whereas this upregulation is missing in TLR-3-/- mice. The TIR domain-containing adaptor inducing IFN-β (TRIF) protein expression showed a similar regulation in wt mice over time. TRIF was significantly upregulated after 24 h of reperfusion compared to 1 or 3 h of reperfusion (54.38±6.35 (24 h) vs. 25.37±2.92 (1 h) resp. 28.79±3.28 (3 h) relative protein expression, P<0.01). In TLR-3-/- mice, TRIF protein expression remained unchanged over time. Additionally, the investigation of the transcriptional status of the TLR-3 activation surrogate interferon (IFN)-β1, a direct target of TRIF and IRF-3 revealed an increased mRNA turnover in wt mice over time (106.4±20.28 (24 h) vs. 17.51±3.77 (1 h) resp. 7.53±0.82 (3 h) relative mRNA expression, P<0.001), whereas TLR-3-/- animals lacked this upregulation. These results indicate that the TLR-3 pathway is activated upon IR in vivo (Figure 1C).

### TLR-3 activation correlated with pro-inflammatory phenotype in the kidney after IR

**Figure 2:**
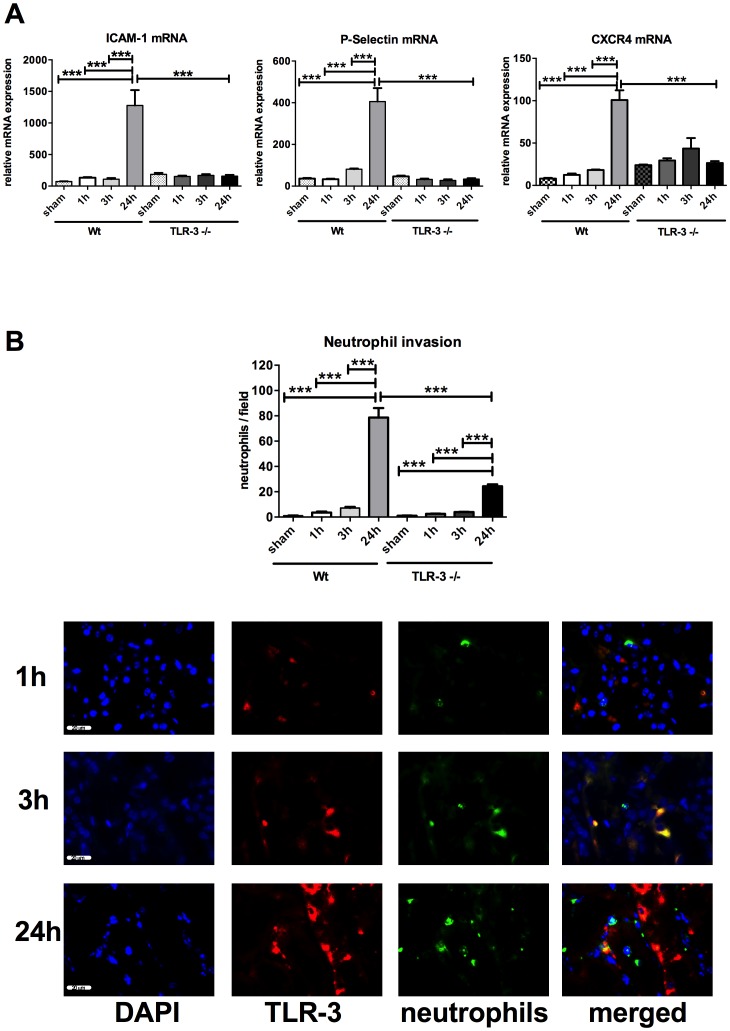
Inflammatory response is higher in wt animals. RT-PCR expression of pro-inflammatory genes ICAM-1, P-Selectin and CXCR4 (A). Neutrophil invasion detected by immunofluorescence (red: TLR-3, green (Alexa 488) (B): neutrophils, blue (DAPI): nuclei. 3 randomly taken pictures from each slide were evaluated using an automatized algorithm (n = 6/group, calibration bar represents 20 µm). (***, P<0.001).

In wt animals, the most significant increase in ICAM-1 (1277±244.1 vs. 72.24±3.34; P<0.0001), P-Selectin (405.60±64.69 vs. 36.27±3.15; P<0.0001) and CXCR4 (100.80±11.54 vs. 8.04±0.91; P<0.0001) gene expression is observed at 24 h vs. the corresponding baselines (shams), whereas in TLR-3-/- mice these levels remained unchanged, indicating a significant lower inflammation level in these animals (Figure 2A). At the level of cellular inflammation, the corresponding neutrophil immunofluorescence staining confirmed the PCR results (Figure 2B). After 24 h, neutrophil invasion was significantly higher in wt mice than in the corresponding TLR-3-/- mice 78.70±7.48 (24 h wt) vs. 24.33±1.47 (24 h TLR-3-/-) neutrophils per field; P<0.001). Again, in both groups neutrophil increase was mainly observed at later stages of reperfusion (24 h). Of note, the extent of cellular inflammation was significantly higher in wt mice than in the TLR-3-/- mice. Interestingly, double staining experiments revealed that the source of TLR-3 derived from renal tubular cells (red) and not from neutrophils (green) (Figure 2B, micrographs).

### TLR-3 activation correlated with tubular damage in wt animals

**Figure 3:**
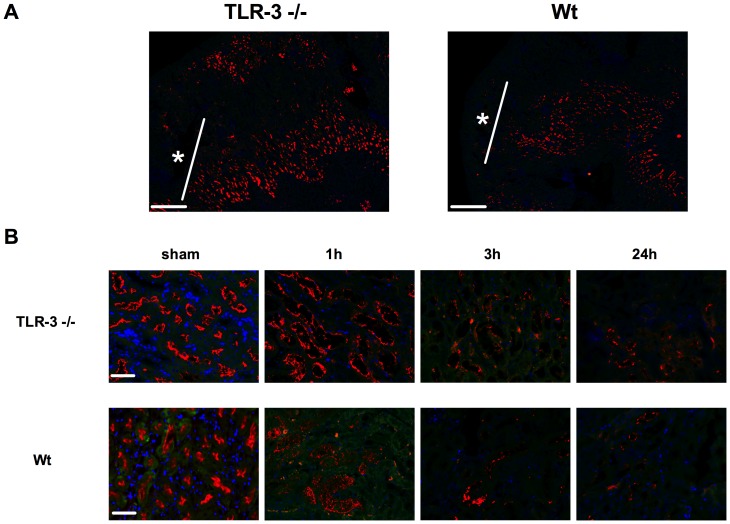
Proximal tubular loss is increased in wt mice. CD13 immunostained kidney tissue (A). White mark determines the outer stripe of the outer medulla* (10× magnification, calibration bar represents 200 µm). Time course of CD13 immunostaining (B). 3 randomly taken pictures from each slide (n = 6/group) were evaluated (red: CD13, blue: DAPI, calibration bar represents 20 µm).

**Figure 4:**
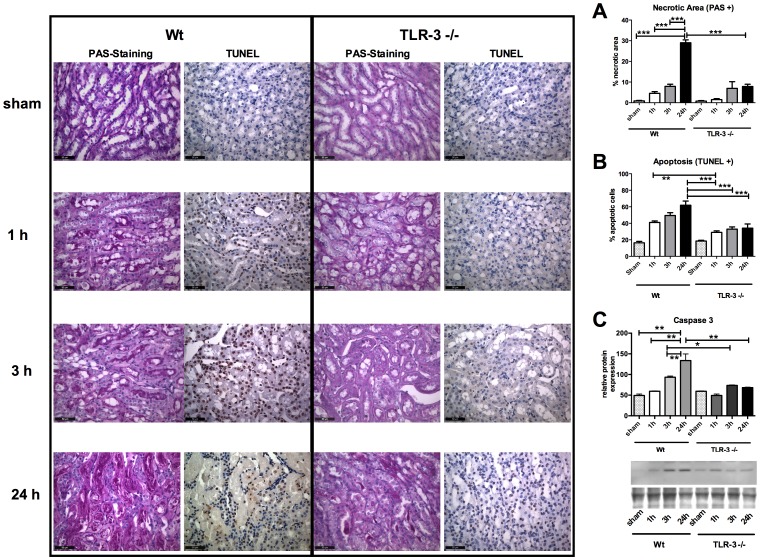
TLR-3 activation induces apoptosis and necrosis. PAS (left micrographs), TUNEL (right micrographs) staining and Caspase-3 western blot analysis (C). PAS staining detects increased necrotic tissue areas in wt animals (A). TUNEL staining detects increased apoptosis in wt animals (B). Western blot analysis detects increased Caspase-3 protein expression in wt animals (C). Automatized evaluation was performed on three randomly assigned pictures from each slide (left: wt mice, right: TLR-3-/- mice; n = 6/group; calibration bar represents 50 µm). (P<0.01; ***, P<0.001).

CD13 specific staining for detection of the tubules revealed that the main damage occurred mainly in the proximal tubules within the outer stripe of outer medulla (* Figure 3A, overview). CD13 expression significantly decreased in wt animals with the duration of reperfusion and showed already significantly reduced CD13 staining after 1 h of reperfusion whereas in TLR-3-/-, CD13 loss only occurred in a mild form after 24 h of reperfusion (Figure 3B). PAS and TUNEL stainings for detection of necrotic and apoptotic cells showed a steady increase in necrosis within the outer stripe of outer medulla over time in wt mice, where the most significant increase occurred in the late stages of reperfusion (0.88±0.23 (baseline) vs. 29.03±1.40 (24 h) % necrotic area; P<0.001) (Figure 4). However, TLR-3-/- mice showed significantly less tissue damage at the end of the observation period compared to the corresponding wt animals (7.84±1.11 (24 h TLR-3-/-) vs. 29.03±1.40 (24 h wt) % necrotic area; P<0.001). Moreover, there was no increase in tissue damage in TLR-3-/- mice. Wt animals also had higher apoptosis rates than the corresponding ko mice. When comparing wt vs. TLR-3-/- mice however, there was a significantly lower apoptosis level in the TLR-3-/- mice (61.89±5.06 (24 h wt) vs. 34.09±5.09 (24 h TLR-3-/-) % apoptotic cells; P<0.001). These data indicate that TLR-3 might also be involved in the induction of proximal tubular damage after IR in the kidney (Figure 4).

### TLR-3-/- mice displayed a better renal perfusion after IR than wt mice

**Figure 5:**
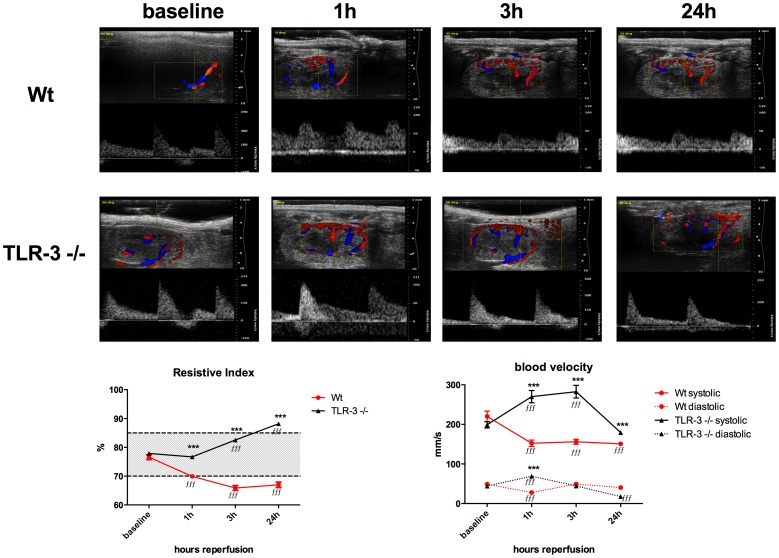
Color duplex sonography. Representative doppler flow profiles before (baseline), 1, 3 and 24 h after ischemia (A). Left graph: resistive index. Dotted lines indicate upper and lower normal limits. Right graph: systolic (upper) and diastolic (lower, dotted) (B). 10 s video sequences from at least 15 flow signature were measured per animal (n = 6/group). Black line: TLR-3-/- mice, red line: wt mice. (ƒƒƒ***, P*<0.001*). ƒ considered statistically different from the corresponding sham and * considered different from the corresponding wt time-point.

We first found that wt animals had an immediate and significant drop in the resistive index (RI) already at 1 h after reperfusion and the RI significantly remained lower compared to pre-operative measurements (76.58±0.83% (baseline) vs. 69.92±0.72% (1 h, P<0.001), 65.88±0.83% (3 h, P<0.001), respectively 66.99±0.42% (24 h, P<0.001)). Interestingly, wt animals had significantly lower RIs at any time point compared to the values in the corresponding TLR-3-/- group. TLR-3-/- animals showed a steady increase in resistive index over time compared to the baseline, whereas only at 24 hours the index was drastically increased (77.88±0.70% (baseline) vs. 76.68±0.51% (1 h, P<0.001), 82.54±0.45 (3 h, P<0.001), respectively 88.19±0.31% (24 h, P<0.001)). There was no difference in the baseline values between wt (76.58%±0.83%) and TLR-3-/- (77.88%±7.70%) animals (Figure 5, left graph).

Secondly, we determined blood velocity as direct parameter for kidney perfusion. We found that wt animals had significantly lower systolic blood velocities during 1 h, 3 h and 24 h of reperfusion as compared to the baseline values (each P<0.001). In wt animals' diastolic blood velocity was only reduced in the acute phase of reperfusion at 1 h compared to baseline (P<0.001). Interestingly, TLR-3-/- mice showed a more important variability in blood velocity over time. Systolic blood velocity significantly increased for 1 h (P<0.001) and 3 h (P<0.001) of reperfusion compared to baseline, whereas at 24 h reperfusion, maximal blood velocity significantly dropped below baseline values (P<0.001). On the other hand, the diastolic blood velocity significantly increased in TLR-3-/- mice at 1 h of reperfusion (P<0.001), and after 24 h of reperfusion, diastolic velocity significantly dropped (P<0.001) below baseline values. When comparing wt vs. TLR-3 animals, we found that systolic blood velocity was in general significantly lower in wt vs. TLR-3-/- mice (P<0.001 at each time point), indicating a lower perfusion in wt animals during reperfusion. These results indicate that TLR-3 is directly involved in the regulation of post ischemic kidney perfusion (Figure 5, right graph).

### Assessment of postischemic renal impairment

**Figure 6:**
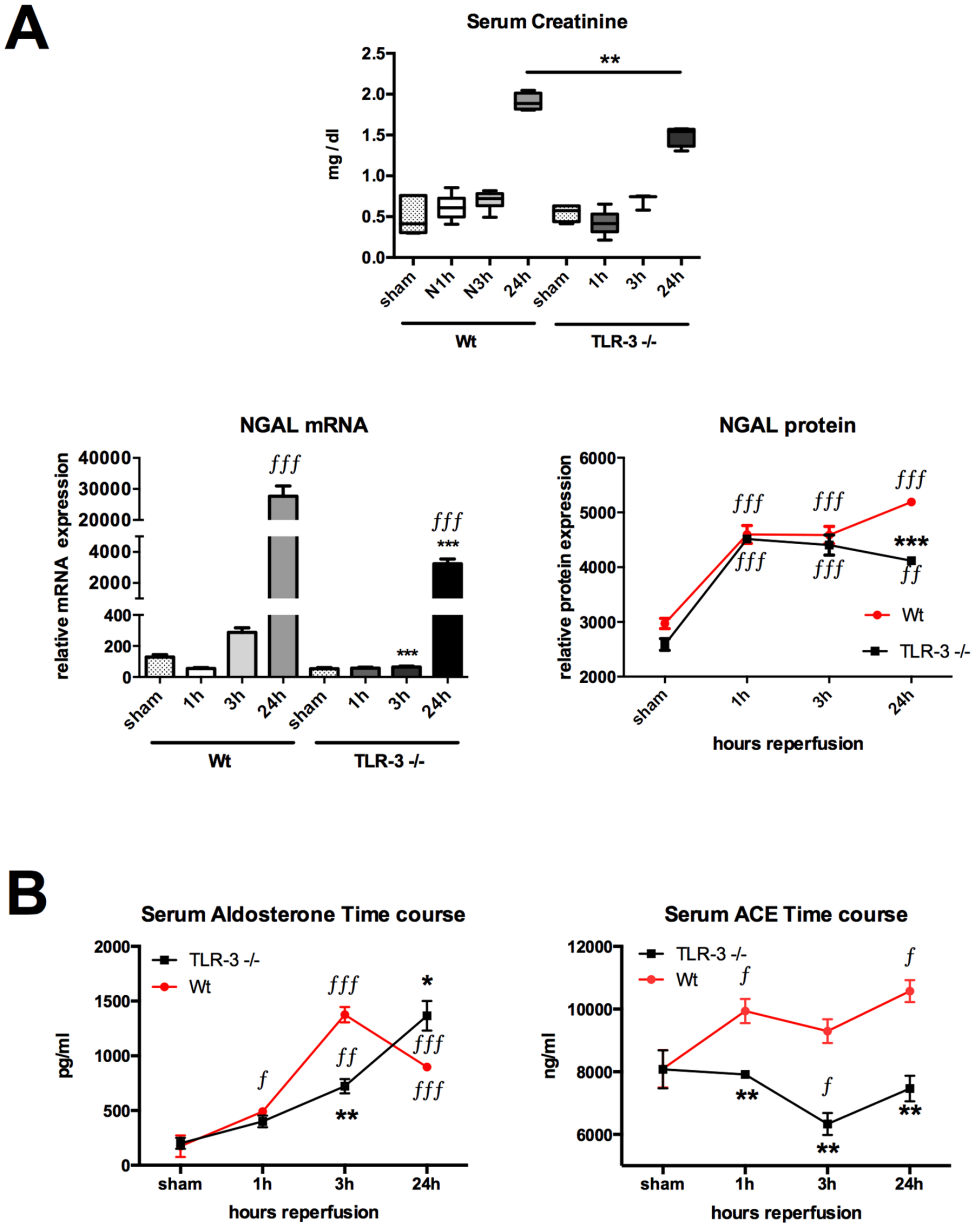
Postischemic renal impairment is stronger in wt animals. Serum creatinine assessment (top) reflecting kidney function (n = 6/group) (A). Messenger RNA (left) and protein (right) expression of the kidney injury marker NGAL (n = 6/group). Determination of serum aldosterone and ACE by ELISA (B). (ƒ*, P<0.05; ƒƒ**, P<0.01; ƒƒƒ***, P<0.001). ƒ considered statistically different from the corresponding sham and * considered different from the corresponding wt time-point.

For postischemic renal function assessment, we first measured serum creatinine. Serum creatinine levels were similar at baseline, 1 h and 3 h after the begin of reperfusion in TLR-3-/- as compared to the corresponding wt (sham:0.5484±0.0529 vs. 0.5086±0.1041 mg/dl; 1 h: 0.4230±0.0617 vs. 0.6150±0.0624 mg/dl; 3 h: 0.06929±0.0566 vs. 0.7014±0.0463 mg/dl; all n.s.). However, after 24, we could observe a significant higher creatinine levels in wt animals as compared to the 24 h TLR-3-/- animals (1.9060±0.0520 vs. 1.4920±0.0626 mg/dl; P<0.01) (Figure 6A, upper graph). It might be, that the gold standard test for kidney function/injury might not be sensitive enough to detect early kidney injury, as creatinine has to accumulate first [25], [26], [27]. For this purpose we also detected Neutrophil gelatinase-associated lipocalin (NGAL), a biomarker of early ischemic acute kidney injury. NGAL was assessed to investigate the renal function. NGAL gene and protein expressions in tissue homogenates were used for kidney function assessment [28], [29]. NGAL gene and protein expressions were significantly induced over time in wt mice, with statistical significance already at 3 h for gene expression followed by marked statistical significance after 24 h of reperfusion for both mRNA and protein expression (24 h vs. sham, 1 and 3 h; P<0.001). NGAL gene expression was likewise increased in TLR-3-/- mice, however the expression level at 24 h was significantly less than in the corresponding wt mice (27,655.0±3323.0 (24 h wt) vs. 3,245.0±299.1 (24 h TLR-3-/-) relative mRNA expression; P<0.001). Here, the difference between wt and TLR-3-/- in NGAL gene expression was nearly 10 fold. NGAL protein rose in both groups similarly, indicating the same ischemic stimulus in both groups. However, after 24 h of reperfusion, NGAL protein levels further increased (5192±9.5 mean pixel densities, P<0.001 vs. 24 h TLR-3-/-) in wt mice whereas in TLR-3-/- mice, NGAL levels started to drop (4118±58.73 mean pixel densities, P<0.001 vs. 24 h wt). (Figure 6A). To strengthen our data about kidney function, we also measured the physiologic markers angiotensin converting enzyme (ACE) and aldosterone (Figure 6B). Aldosterone levels were similar in TLR-3-/- (200.60±43.57 pg/ml) as well as in wt (174.20±9.77 pg/ml) populations at baseline (shams, n.s.). Aldosterone serum levels were quickly upregulated after 1 h in wt animals as compared to the corresponding shams (491.30±30.93 vs. 174.20±9.77 pg/ml; P<0.05) whereas this was not the case in the TLR-3-/- animals (400.10±50.27 vs. 200.6±43.57 pg/ml; n.s.). In the ko mice, aldosterone only started to raise after 3 h (722.50±65.61 vs. 200.60±43.57 pg/ml; P<0.0001) and reached a maximum after 24 h (1365.00±138.00 vs. 200.60±43.57 pg/ml; P<0.0001) as compared to their sham animals. However aldosterone levels were still higher in wt vs. shams at 3 and 24 hours (1376.00±70.32 and 896.70±30.93 vs. 174.20±9.77 pg/ml; P<0.0001 each), its levels started to drop at 24 hours, which was not the case in the TLR-3-/- animals, where the levels still increased (896.70±30.93 vs. 1365.00±138.00 pg/ml; P<0.0001) (Figure 6B, left graph). ACE levels were comparable at the baseline between the TLR-3-/- and wt mice (8777.00±664.30 vs. 8095.00±597.10 ng/ml; n.s.). Moreover, we could only observe an increase in ACE in the wt animals at 1 h (9936.00±386.70 vs. 8095.00±597.10 ng/ml; P<0.05) and 24 h (10574.00±350.70 vs. 8095.00±597.10 ng/ml; P<0.05) as compared to the shams, whereas ACE levels in the TLR-3-/- mice remained stable over time. Except for the 3 h time-point, where a drop in ACE levels could be observed in the TLR-3-/- mice vs. their shams (6331.00±351.80 vs. 8777.00±664.30 ng/ml; P<0.05) (Figure 6B, right graph).

### Endothelial turnover

**Figure 7:**
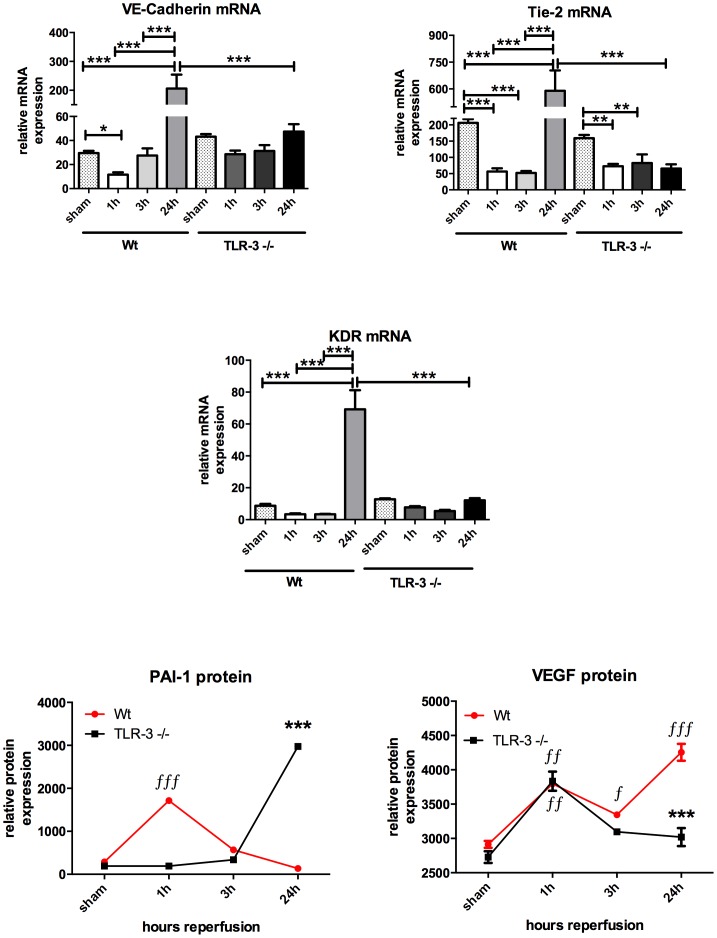
Endothelial turnover. Gene expression profiles of endothelial relevant genes were evaluated to analyze endothelial turnover. VE-cadherin, Tie-2 and KDR mRNA expression was analyzed using RT-PCR, PAI-1 and VEGF protein expression was analyzed using a protein array. PCR analyses were performed in triplicates and for protein analyses, pooled samples from each group was measured in duplicates. Columns and error bars represent means ± SEM; red line represents the time-course of PAI-1 and VEGF in wt animals and black line corresponds to the TLR-3 ko mice; ∫*, P<0.05, **, P<0.01 and ∫∫∫***, P<0.001; one-way ANOVA and Bonferroni's multiple comparison test. ƒ considered statistically different from the corresponding sham and * considered different from the corresponding wt time-point.

*In vivo* endothelial turnover was assessed by using VE-cadherin, Tie-2 as well as KDR (VEGF-R2) gene expression as well as plasminogen activator inhibitor (PAI)-1 and VEGF as surrogates [24], [30]. Gene expression of VE-cadherin, a molecule that is substantial for endothelial coherence, drop after 1 h reperfusion and then increased significantly after 24 h vs. baseline in wt animals. Tie-2 as well as VEGF-receptor 2 (KDR) gene expressions behaved the same way like VE-cadherin by first dropping after 1 h of reperfusion and then increasing significantly after 24 h of reperfusion. However, in TLR-3 ko mice a distinct endothelial loss was also observed in the early reperfusion phase when looking at Tie-2 mRNA data (159.10±9.96 (sham) vs. 72.84±7.15 (1 h); P<0.001). Contrary to these molecules, the plasminogen activator inhibitor (PAI)-1, a natural inhibitor of endothelial activation is only increased in TLR-3 ko mice, indicating a lesser extent of activation in these animals and correlating with the lower cellular inflammation occurring in the TLR-3 animals. Accordingly VEGF, as most potent stimulator of angiogenesis, first increased significantly in both groups compared to the corresponding baseline values. Hence, after 24 hours of reperfusion, VEGF protein significantly dropped in TLR-3-/- vs. wt mice (3019±132.4 vs. 4256±123.3 mean pixel densities, P<0.001) (Figure 7).

## Discussion

Extensive research has been performed in the last decades to elucidate the molecular mechanisms underlying acute kidney injury (AKI). To date we know that TLRs are involved in the pathogenesis of ischemia and reperfusion-related inflammation and AKI [31], [32], [33], [34], [35]. However, TLR-3 has not been described in renal IR so far. Here we demonstrate that the TLR-3 receptor system is rapidly induced upon IR, already within 1–3 hours and correlates with the severity of AKI.

Among various examined organs, we observed TLR-3 to be most strongly expressed in the kidneys, underlining its relevance in renal tissue signaling (Figure 1A). The idea that especially TLR-3 is involved in AKI, is rooted by the hypothesis that upon cellular injury, cytoplasmic nucleotides (i.e. RNA) may be liberated which might then activate neighboring cells and hence induce inflammation. The fact that in our model TLR-3 positive cells were not neutrophils but probably adjacent tubular cells (Figure 2B) strengthens our hypothesis.

Before analyzing the data, we aimed to know whether the ischemic hit in both mouse populations were identical. For this purpose, we measured three surrogates for hypoxia, namely hypoxia inducible factor (HIF)-1 α mRNA, CA IX mRNA (Text S1, Figure S1 A, B) and vascular endothelial growth factor (VEGF) A protein expression (Figure 7). Both molecules are direct hypoxia markers and are upregulated within minutes following hypoxia [22]. We could show that the ischemic hit was similar in both mouse populations. Interestingly, VEGF protein levels still rose in wt animals as expression of an ongoing endothelial disorder. Specific CD13 staining for detection of the proximal tubules revealed that the most important damage occurs in the proximal tubular system within the outer stripe of outer medulla (Figures 3,4). Additional PAS stainings of the renal cortex revealed that in this region there were no significant changes concerning cellular damage between the groups and at any time point (Text S2, Figure S2). To test whether kidney function might already be impaired after 24 h of reperfusion, we measured serum creatinine and we could show, that creatinine was significantly higher in the wt as in the TLR-3-/- mice after 24 h. However, the levels in the ko mice were still increased as compared to the baselines, which is due to other mechanisms that might rescue the TLR-3 pathway only later (at 24 h only), as for example other TLR pathways. Leemans as well as Wu and colleagues could show, that for example TLR-2/-4 play a role in the development of kidney I/R, but only after 1 to 10 days post ischemia [13], [15]. We think in contrast, that TLR-3 might represent an early trigger mechanism that might stand in a cross talk with other TLRs, which play a role in kidney I/R [36], [37], [38]. Therefore one big concern was the reliable analysis reflecting also very early kidney dysfunction after 1 h and 3 h. As the gold standard, serum creatinine, displays poor specificity and sensitivity with regard to this early period of acute kidney injury [25], [26], [27] and taking into account that TLR-3 mediated signaling initiates already within the first hours after IR, we also explored NGAL a novel very early diagnostic and prognostic biomarker of AKI [25], [26], [27], [29]. NGAL quickly increased in both groups after 1 h, reflecting the initial ischemic hit. However, in TLR-3-/- mice, NGAL levels plateaued at 3 h and even slightly dropped at 24 h, whereas the levels steadily increased in wt animals (Figure 6). Another marker, namely angiotensin converting enzyme (ACE), that is upregulated when blood pressure drops below a physiologic level [39], is increased in wt animals (Figure 6). This goes hand in hand with the observations regarding the resistive index, where we showed that wt animals suffered from vasoplegia (Figure 5). Accordingly, the low ACE levels in the TLR-3 ko mice corresponded with higher RI's and thus lower renal damage. The mineralocorticoid aldosterone which acts directly on the collecting duct of the kidney by increasing water reabsorption is increased in both groups over time, indicating a volume misbalance (Figure 6). However, the increase is faster in wt as in TLR-3 ko mice, indicating a much lower degree of AKI in the ko mice. Interestingly, after 24 h reperfusion, aldosterone still increased in the ko mice (which is in concordance with the RI observations and the increased blood velocity) (Figure 5,6). As high aldosterone levels have been observed with enhanced vasodilatation in animal models, it might represent a mechanism to counteract the increase in vascular resistance occurring in the TLR-3 ko animals [40].

Changes in blood flow or perfusion also implicate endothelial dysfunction or damage, which we also observed in our model where the wt mice showed a high endothelial turnover (Figure 7) and also a higher extent of inflammation and recruitment of neutrophils enhancing the tubular damage (Figure 2 A, B). The fact that in the wt animals TNF-α behaves completely contrary to the ko mice (Text S3, Figure S3) is plausible, as we already demonstrated that the pathologic hit was so strong in both groups and that an initial inflammatory reaction occurred, probably independent of TLR-3. The reason why early inflammation is attenuated in the ko mice might be explained by the fact, that classical pro-inflammatory proteins such as IL-1α, IL-17, IL-27 and MIP-1β have significantly lower baseline levels in TLR-3-/- mice as in the wild-types. Nevertheless the interpretation of these data remains speculative at the moment and further studies are needed to identify the exact molecular players. This difference in protein expression is at least plausible, as these molecules are TLR-3 targets (Text S3, Figure S3) [41], [42], [43], [44], [45].

The natural occurring inhibitor of inflammation, tissue inhibitor of metalloproteinase (TIMP)-1 is also increased in the TLR-3-/- mice, strengthening our hypothesis that TLR-3 might suppress anti-inflammatory mechanisms under normal conditions (Text S3, Figure S3). Finally, our hypothesis is further sustained by the observation that in the wt animals, apoptosis and necrosis is drastically increased whereas in the TLR-3-/- mice this process is significantly alleviated.

Taken together our findings strengthen the notion that TLR-3 is involved in renal IR injury. We hypothesize that its activation might be induced by liberation of TLR-3 ligands by apoptotic cells. Whether the liberation of the ligands is occurring from the proximal tubular cells or whether also cells of the hematopoietic lineage are involved in nucleotide liberation remains unclear at the moment and is subject of further studies. Although TLR-3 activation represents one pathway involved in AKI following renal IR, it though seems to occupy an important role in triggering and enhancing early inflammatory processes in proximal tubules.

## Supporting Information

Figure S1 **Ischemic hit is similar in both mouse populations.** Gene expressions of Hypoxia Inducible Factor (HIF)-1 (A) and Carbo-anhydrase (CA)IX (B). (ƒƒƒ***, P<0.001). ƒ considered statistically different from the corresponding sham and * considered different from the corresponding wt time-point.
(TIFF)

Figure S2 **The renal cortical region is not affected by IR.** Cortical PAS staining. Automatized evaluation was performed on three randomly assigned pictures from each slide (left: wt mice, right: TLR-3-/- mice; n = 6/group; calibration bar represents 50 µm).
(TIFF)

Figure S3 **Inflammation is differentially regulated.** Expression of TNF-α, TIMP-1 (A) and the pro-inflammatory mediators IL-1a, MIP-1β, IL-17 and IL-27 (B). Samples were pooled according the time-point and group (red: wt, black: TLR-3-/-). (ƒ*, P<0.05; ƒƒ**, P<0.01; ƒƒƒ***, P<0.001). ƒ considered statistically different from the corresponding sham and * considered different from the corresponding wt time-point.
(TIFF)

Text S1 **Ischemic hit is similar in both mouse populations.** To check whether the two populations were subjected to the same intensity of ischemia, we measured the hypoxia surrogates hypoxia inducible-factor (HIF)-1 α and Carbo-anhydrase (CA) IX.
(DOC)

Text S2 **Cell damage upon IR is not affecting the cortical area.**
(DOC)

Text S3 **Inflammation is differentially regulated in wt and TLR-3-/- mice.** To analyze inflammation regulation, we detected protein levels of classical mediators of inflammation such as tumor necrosis factor (TNF)-α, tissue inhibitor of metalloproteinase (TIMP)-1, IL-1a, macrophage inflammatory protein (MIP)-1β, IL-17 and IL-27.
(DOC)
