# Supplementary figures and images for: The Early Activation of Toll-Like Receptor (TLR)-3 Initiates Kidney Injury after Ischemia and Reperfusion
Source: PLoS One. 2014 Apr 15;9(4):e94366. doi: 10.1371/journal.pone.0094366 (PMC3988056; doi:10.1371/journal.pone.0094366)

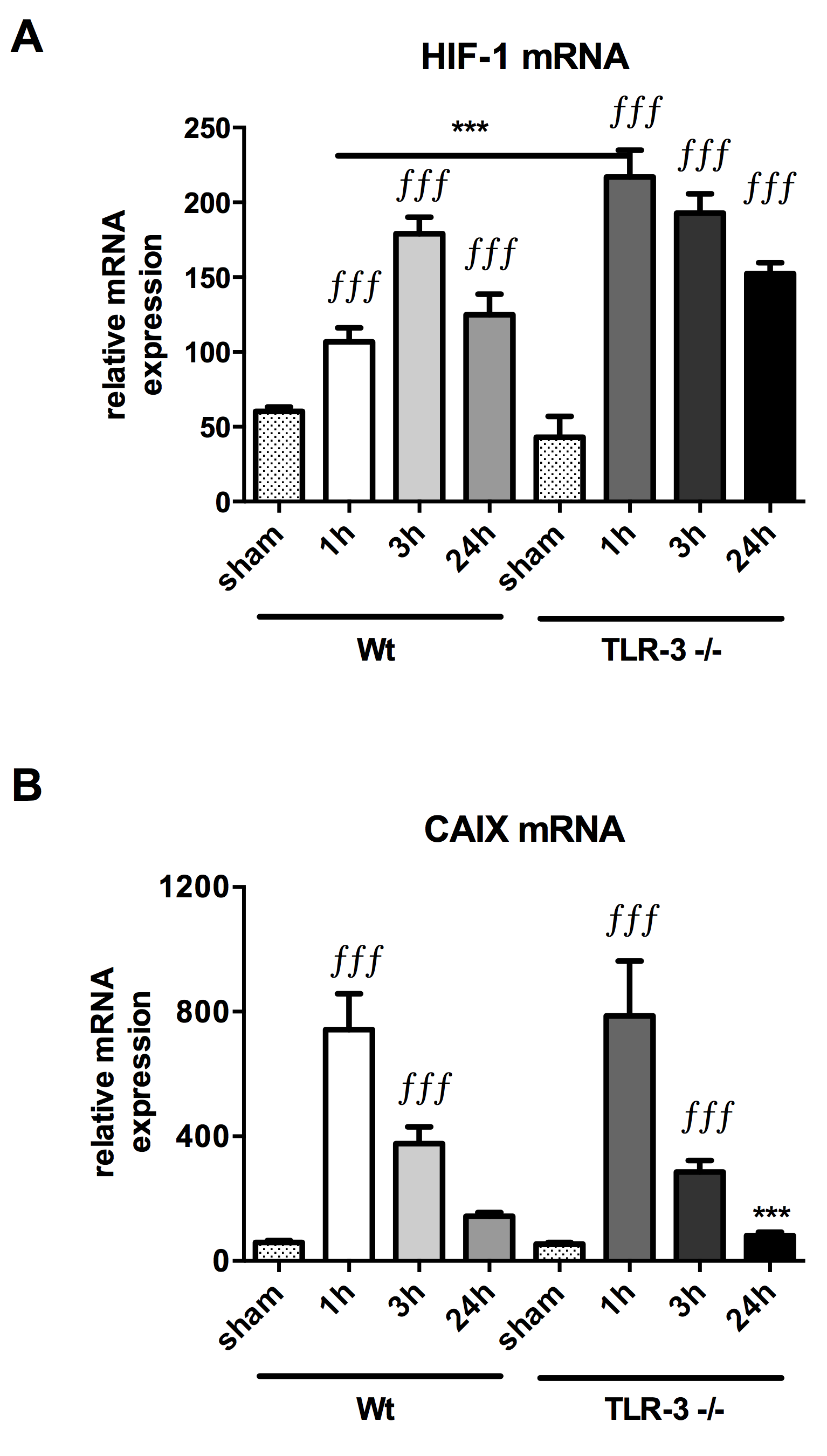

Supplement: Figure S1 — Ischemic hit is similar in both mouse populations. Gene expressions of Hypoxia Inducible Factor (HIF)-1 (A) and Carbo-anhydrase (CA)IX (B). (ƒƒƒ***, P<0.001). ƒ considered statistically different from the corresponding sham and * considered different from the corresponding wt time-point. (TIFF) [file pone.0094366.s001.tiff]

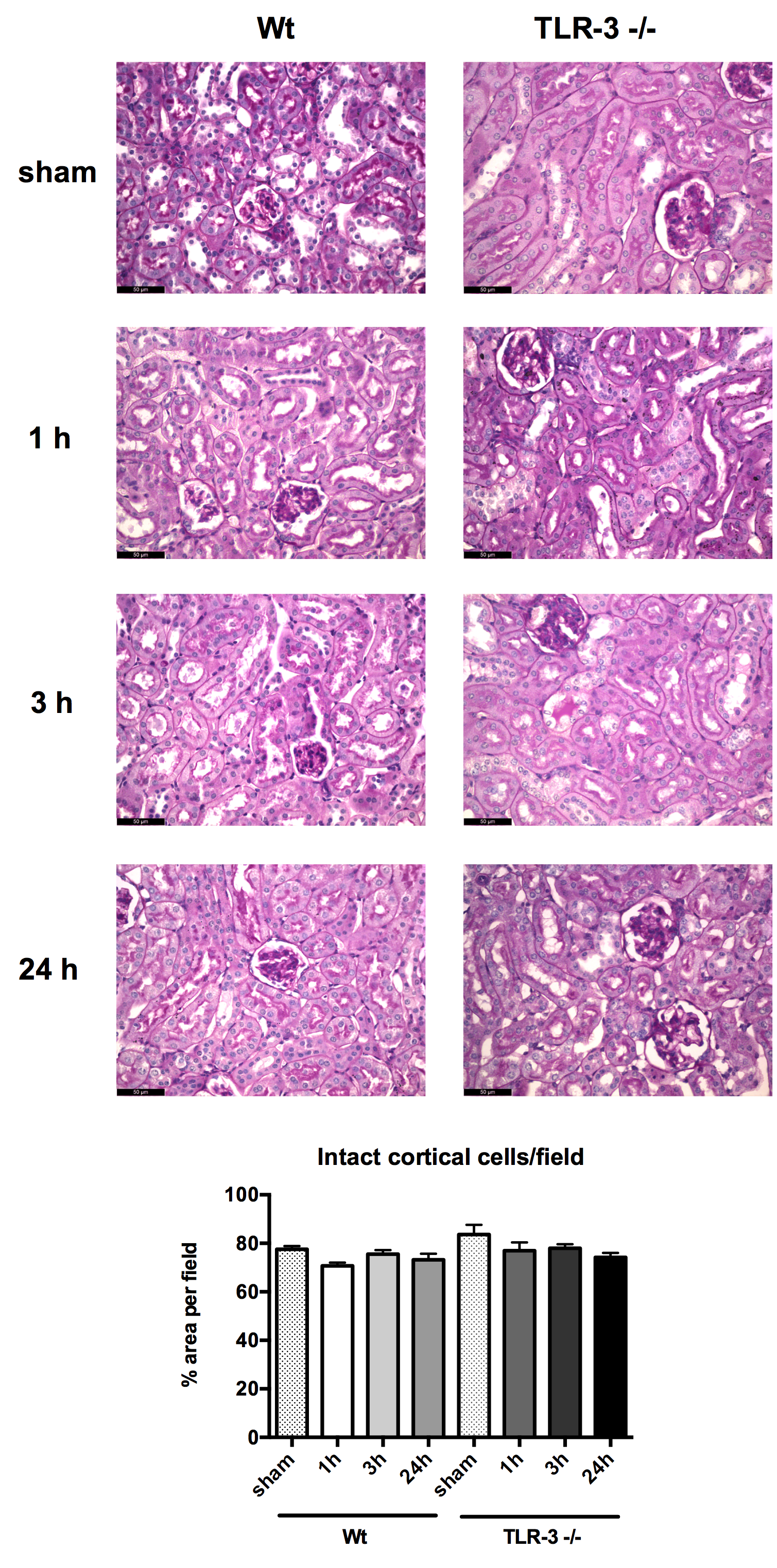

Supplement: Figure S2 — The renal cortical region is not affected by IR. Cortical PAS staining. Automatized evaluation was performed on three randomly assigned pictures from each slide (left: wt mice, right: TLR-3-/- mice; n = 6/group; calibration bar represents 50 µm). (TIFF) [file pone.0094366.s002.tiff]

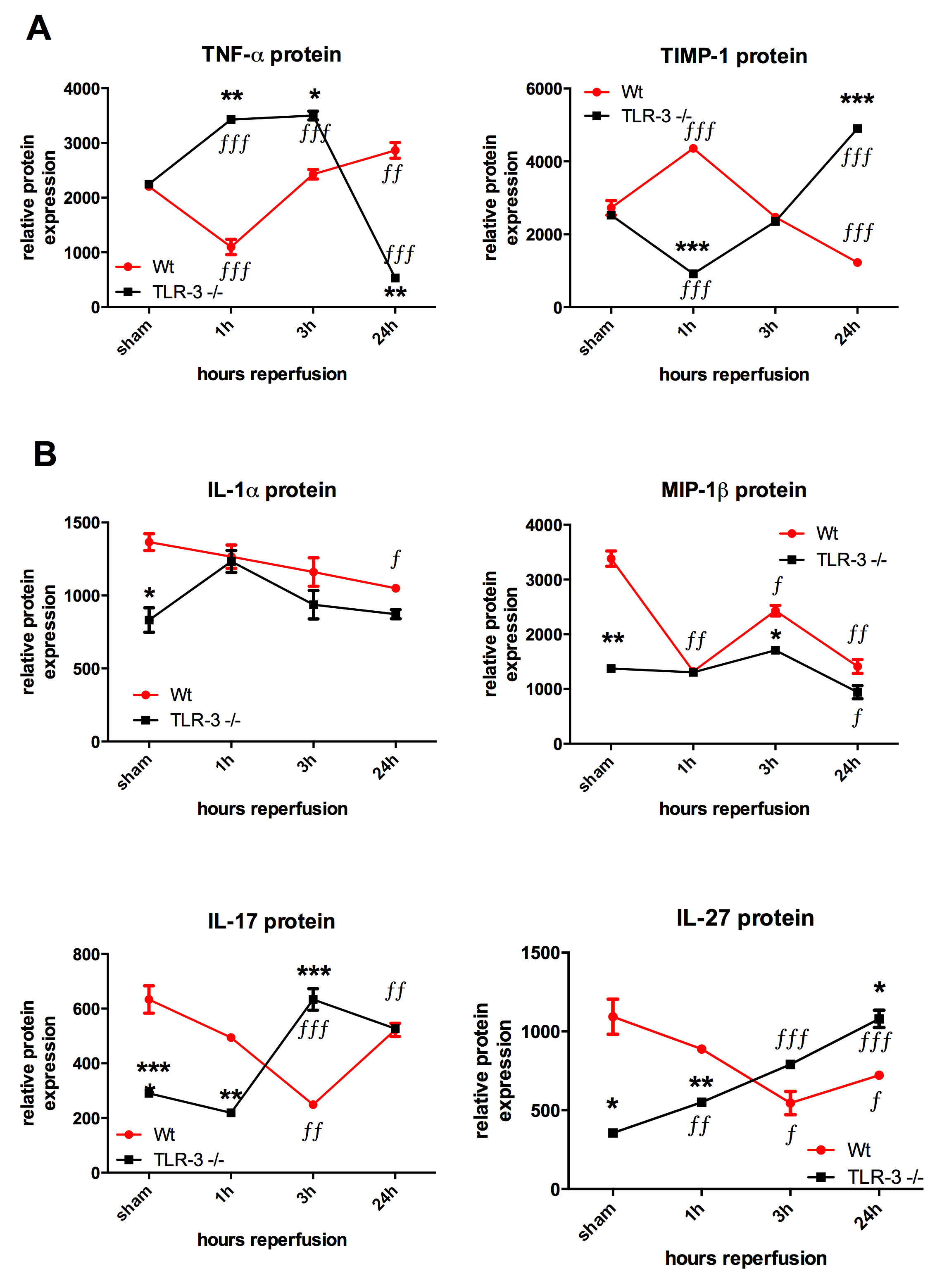

Supplement: Figure S3 — Inflammation is differentially regulated. Expression of TNF-α, TIMP-1 (A) and the pro-inflammatory mediators IL-1a, MIP-1β, IL-17 and IL-27 (B). Samples were pooled according the time-point and group (red: wt, black: TLR-3-/-). (ƒ*, P<0.05; ƒƒ**, P<0.01; ƒƒƒ***, P<0.001). ƒ considered statistically different from the corresponding sham and * considered different from the corresponding wt time-point. (TIFF) [file pone.0094366.s003.tiff]
