# Supplementary material for: The Early Activation of Toll-Like Receptor (TLR)-3 Initiates Kidney Injury after Ischemia and Reperfusion
Source: PLoS One. 2014 Apr 15;9(4):e94366. doi: 10.1371/journal.pone.0094366 (PMC3988056; doi:10.1371/journal.pone.0094366)
Supplement: Text S1 — Ischemic hit is similar in both mouse populations. To check whether the two populations were subjected to the same intensity of ischemia, we measured the hypoxia surrogates hypoxia inducible-factor (HIF)-1 α and Carbo-anhydrase (CA) IX. (DOC) [file pone.0094366.s004.doc]

**Supporting Information 1. Ischemic hit in both mouse populations is similar.**

To analyze whether both mouse populations (TLR-3-/- and wt) were subjected to the same extent of ischemia, we measured gene expression of two surrogate markers of hypoxia. The hypoxia inducible-factor (HIF)-1 α is a pivotal molecule that is involved in most hypoxia driven pathways. HIF-1 mRNA expression was significantly upregulated in TLR-3-/- (217.00 ± 17.94 vs. 43.06 ± 13.98; P<0.0001) as well as wt (106.80 ± 9.36 vs. 60.38 ± 2.80;P<0.0001) populations after 1 hour as compared to the corresponding sham baselines. When comparing HIF-1 mRNA expressions after 1h, we could observe a significantly higher expression in TLR-3-/- compared to the corresponding wt mice (217.00 ± 17.94 vs. 106.80;P<0.0001), whereas the HIF-1 mRNA expression equalized after 3h and 24h (Figure S1 A).

Carbo-anhydrase (CA) IX is a molecule that is a target for HIF-1 and that is selectively and quickly upregulated by the latter . CAIX may thus serve as surrogate for HIF-1 downstream activity. We observed that CAIX mRNA expression was significantly upregulated in TLR-3-/- (742.10 ± 115.90 vs. 59.63 ± 5.63; P<0.0001) and wt (786.00 ± 176.10 vs. 53.11 ± 5.10; P<0.0003) after 1h when compared to the corresponding sham baselines. CAIX mRNA expressions were similar in wt vs. TLR-3-/- mice at 1h and 3h of reperfusion indicating that HIF-1 activity and thus hypoxic stimulus was similar in both populations. However, CAIX dropped faster, almost to normal values in TLR-3-/- mice vs. wt after 24 h reperfusion (P<0.001) (Figure S1 B)

**References**

1. Paulus P, Ockelmann P, Tacke S, Karnowski N, Ellinghaus P, et al. (2012) Deguelin attenuates reperfusion injury and improves outcome after orthotopic lung transplantation in the rat. PLoS One 7: e39265.
