# Supplementary material for: The Early Activation of Toll-Like Receptor (TLR)-3 Initiates Kidney Injury after Ischemia and Reperfusion
Source: PLoS One. 2014 Apr 15;9(4):e94366. doi: 10.1371/journal.pone.0094366 (PMC3988056; doi:10.1371/journal.pone.0094366)
Supplement: Text S2 — Cell damage upon IR is not affecting the cortical area. (DOC) [file pone.0094366.s005.doc]

**Supporting Information 2. Cell damage upon IR is not affecting the cortical area.**

To analyze whether the cortical area is also subjected to damage upon IR, we performed a PAS staining of these regions to detect necrotic areas. After quantification of the amount of intact cells using the automatized MatLab algorithm, we observed that there was no difference between the groups, indicating that there is no significant damage occurring in the cortex following IR (Figure S2).
