# Supplementary material for: The Early Activation of Toll-Like Receptor (TLR)-3 Initiates Kidney Injury after Ischemia and Reperfusion
Source: PLoS One. 2014 Apr 15;9(4):e94366. doi: 10.1371/journal.pone.0094366 (PMC3988056; doi:10.1371/journal.pone.0094366)
Supplement: Text S3 — Inflammation is differentially regulated in wt and TLR-3-/- mice. To analyze inflammation regulation, we detected protein levels of classical mediators of inflammation such as tumor necrosis factor (TNF)-α, tissue inhibitor of metalloproteinase (TIMP)-1, IL-1a, macrophage inflammatory protein (MIP)-1β, IL-17 and IL-27. (DOC) [file pone.0094366.s006.doc]

**Supporting Information 3. Inflammation is differentially regulated in wt and TLR-3-/- mice.**

TNF-α protein expression was higher at 1h and 3h of reperfusion when compared to the corresponding wt time-points (P<0.01 for 1h and P<0.05 for 3h). Only after 24 h of reperfusion, TNF-α protein levels significantly dropped vs. the corresponding wt mice and even below baseline values of the TLR-3-/- mice (P<0.001) (Figure S3A). Concerning the anti-inflammatory TIMP-1, its protein expression after 1h was significantly higher in wt vs. the corresponding ko mice. However, TIMP-1 protein expression quickly dropped over time, so that after 3h the expression levels did not differ and after 24 h TIMP-1 was significantly overexpressed in the ko mice (P<0.001) (Figure S3A). The levels of the pro-inflammatory mediators IL-1a, MIP-1β, IL-17 and IL-27 already differed significantly at the baseline level. In general, these mediators were already significantly higher in the wt mice. IL-1a, MIP-1β and IL-27 dropped over time in the wt mice whereas in the ko animals an increase for IL-17 (P<0.001), IL-27 (P<0.001) and a decrease over time (after 24h) of MIP-1β (P<0.05) could be observed (Figure S3B).
